# Histidine phosphorylation of NME1 regulates the Hippo pathway via the ARHGAP17–CDC42–cytoskeleton axis

**DOI:** 10.1093/lifemedi/lnag002

**Published:** 2026-03-12

**Authors:** Xian Liu, Zhongnan Chen, Jianxi Zhu, Shengcheng Deng, Zhifen Zhou, Wenbin Ma, Zhou Songyang

**Affiliations:** MOE Key Laboratory of Gene Function and Regulation, State Key Laboratory of Biocontrol, Guangzhou Key Laboratory of Healthy Aging Research, School of Life Sciences, Institute of Healthy Aging Research, Sun Yat-sen University, Guangzhou 510275, China; MOE Key Laboratory of Gene Function and Regulation, State Key Laboratory of Biocontrol, Guangzhou Key Laboratory of Healthy Aging Research, School of Life Sciences, Institute of Healthy Aging Research, Sun Yat-sen University, Guangzhou 510275, China; MOE Key Laboratory of Gene Function and Regulation, State Key Laboratory of Biocontrol, Guangzhou Key Laboratory of Healthy Aging Research, School of Life Sciences, Institute of Healthy Aging Research, Sun Yat-sen University, Guangzhou 510275, China; MOE Key Laboratory of Gene Function and Regulation, State Key Laboratory of Biocontrol, Guangzhou Key Laboratory of Healthy Aging Research, School of Life Sciences, Institute of Healthy Aging Research, Sun Yat-sen University, Guangzhou 510275, China; Innovative Center of Health, Longevity and Synthetic Biology, Hainan Academy of Medical Sciences, Hainan Medical University, Haikou 571199, China; Sun Yat-sen Memorial Hospital, Sun Yat-sen University, Guangzhou 510120, China; MOE Key Laboratory of Gene Function and Regulation, State Key Laboratory of Biocontrol, Guangzhou Key Laboratory of Healthy Aging Research, School of Life Sciences, Institute of Healthy Aging Research, Sun Yat-sen University, Guangzhou 510275, China; MOE Key Laboratory of Gene Function and Regulation, State Key Laboratory of Biocontrol, Guangzhou Key Laboratory of Healthy Aging Research, School of Life Sciences, Institute of Healthy Aging Research, Sun Yat-sen University, Guangzhou 510275, China; Innovative Center of Health, Longevity and Synthetic Biology, Hainan Academy of Medical Sciences, Hainan Medical University, Haikou 571199, China; Sun Yat-sen Memorial Hospital, Sun Yat-sen University, Guangzhou 510120, China

## Abstract

**NME1 is a key metastasis suppressor whose activity depends on histidine phosphorylation, yet the biological significance of this modification remains poorly understood. Here, we reveal a previously unrecognized role for NME1 in regulating the Hippo pathway. Using PhastID-based proximity labeling combined with functional assays, we demonstrate that NME1 modulates CDC42 activity via ARHGAP17, a GTPase-activating protein, thereby influencing cytoskeletal organization and Hippo activation. Loss of NME1 reduced YAP phosphorylation and promoted its nuclear localization, indicating suppression of Hippo signaling. These findings define a histidine phosphorylation-dependent NME1–ARHGAP17–CDC42–cytoskeleton axis that controls the Hippo pathway, providing new insights into the functional repertoire of NME1 in cancer and development**.

## Introduction

Histidine phosphorylation is an essential post-translational modification in prokaryotes, lower eukaryotes, and plants, serving as a key intermediate in two-component signaling systems. However, in mammalian cells, histidine phosphorylation remains a relatively underexplored modification due to its chemical instability and the lack of widely available detection methods [1]. Among the known histidine kinases, the *NM23* (non-metastatic 23) gene family, particularly *NME1* (also known as *NM23-H1*), has been extensively studied for its role in tumor metastasis suppression [2].

The human NME/NDPK (Nucleoside Diphosphate Kinase) family comprises 10 members, with NME1 and its paralog NME2 recognized as the primary histidine kinases in human cells [3]. Beyond their nucleoside diphosphate kinase (NDPK) activity, these proteins exhibit histidine kinase function and have been implicated in diverse biological processes, including nucleotide metabolism, cytoskeletal organization, and signal transduction [4–7]. Despite extensive research on NME1’s role in metastasis suppression, the biological relevance of its histidine phosphorylation activity and its downstream signaling mechanisms remain largely undefined.

The Hippo pathway is a highly conserved signaling cascade that controls organ size, cell proliferation, and apoptosis [8, 9]. Central to this pathway are the mammalian sterile 20-like kinase 1 and 2 (MST1/2) and LATS1/2 kinases (Large tumor Suppressor Kinase 1 and 2), which regulate the activity and subcellular localization of the transcriptional co-activators Yes-associated protein (YAP) and transcriptional co-activator with PDZ-binding motif (TAZ) [10, 11]. Dysregulation of Hippo signaling, particularly YAP hyperactivation, is frequently observed in various cancers, contributing to uncontrolled cell growth and metastasis [12]. Upstream regulation of the Hippo pathway is influenced by cytoskeletal dynamics and small Rho GTPases, including CDC42 (Cell Division Cycle 42), which modulates actin organization and has been shown to affect YAP/TAZ activity [13–15].

Given NME1’s role in cytoskeletal organization and its function as a histidine kinase, we hypothesized that NME1 may intersect with Hippo pathway regulation through cytoskeletal modulators. In this study, we employed PhastID-based proximity labeling [16] to map the protein interaction networks of NME1 and investigated its functional relationship with the Hippo pathway. We identified ARHGAP17 (Rho GTPase Activating Protein 17), a CDC42-specific GTPase-activating protein, as a critical mediator linking NME1 histidine phosphorylation to CDC42 activity and Hippo pathway modulation. Our findings reveal a previously unrecognized role for NME1 in modulating the Hippo signaling pathway through a histidine phosphorylation-dependent ARHGAP17–CDC42–cytoskeleton axis, providing new insights into the mechanistic repertoire of NME1 in cancer progression and development.

## Results

### PhastID identifies NME1 histidine kinase-dependent interactors

**Figure 1.**
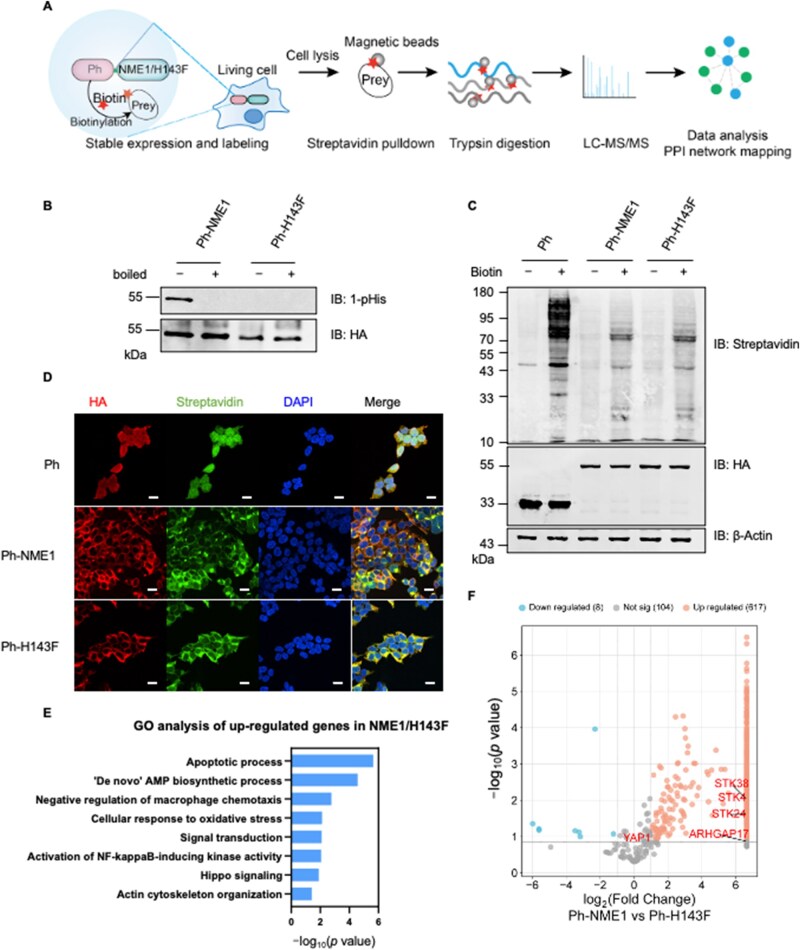
Application of PhastID to identify NME1 HK dependent interactors. (A) Schematic diagram of the Ph-NME1/H143F PhastID workflow. (B) Flag immunoprecipitation of lysates from HEK293T cells overexpressing Flag-HA-tagged Ph-NME1/H143F. Eluents were treated with or without 95°C for 10 min, followed by detection of N1 histidine phosphorylation using anti-1-pHis antibody. Total overexpressed proteins were probed with anti-HA antibodies. (C) Detection of expression and biotin labeling in HEK293T cells expressing Ph, Ph-NME1, or Ph-H143F, with or without 50 μmol/L biotin treatment for 16 h. Cell lysates were separated by SDS-PAGE, and biotin signals were detected using streptavidin antibodies. Anti-HA and anti-β-actin antibodies served as loading controls. (D) Immunofluorescence analysis of subcellular localization and biotin labeling of Ph, Ph-NME1, and Ph-H143F after 16 h of biotin treatment. HA signal (red) indicates target protein, streptavidin (green) indicates biotinylation, and DAPI (blue) marks nuclei. Merged images show colocalization (scale bar: 20 μm). (E) GO analysis of upregulated genes in Ph-NME1 compared to Ph-H143F. Biological pathway clustering was performed using the Database for Annotation, Visualization and Integrated Discovery (DAVID) database. (F) Volcano plot displaying differentially expressed genes in Ph-NME1 vs. Ph-H143F. Genes of interest are highlighted in red. This figure was generated using the Weishengxin platform.

To investigate the histidine phosphorylation-dependent interactome of NME1, we performed PhastID-based proximity labeling in HEK293T cells expressing either wild-type NME1 (Ph-NME1) or its histidine kinase-deficient mutant (Ph-H143F) (Fig. 1A). Mass spectrometry data for Ph-NME1 and Ph-H143F are available in Table S2. Western blot analysis using an N1-phosphohistidine-specific antibody [17] confirmed strong histidine phosphorylation in Ph-NME1, whereas Ph-H143F lacked detectable phosphorylation, validating its kinase-inactive status (Fig. 1B). To control for non-specific biotinylation, we included cells expressing PhBPL alone (hereafter referred to as “Ph”) as a negative control. The efficiency of biotin labeling was confirmed by streptavidin immunoblotting, showing clear biotinylation signals in Ph, Ph-NME1, and Ph-H143F upon biotin supplementation, with consistent expression confirmed by HA immunoblotting (Fig. 1C). Immunofluorescence further demonstrated robust biotin labeling and cytoplasmic distribution of Ph, Ph-NME1, and Ph-H143F, confirming effective proximity labeling and expression in cells (Fig. 1D).

To explore potential pathways regulated by NME1 histidine phosphorylation, Gene Ontology (GO) enrichment analysis of upregulated proteins in the Ph-NME1 interactome revealed significant enrichment in pathways related to actin cytoskeleton organization and Hippo signaling (Fig. 1E). Consistently, a volcano plot highlighted significant upregulation of Hippo pathway components, including STK38, STK4, and STK24, in the Ph-NME1 group compared to the kinase-inactive Ph-NME1-H143F (Fig. 1F). These findings suggest that NME1 modulates the Hippo pathway in a histidine phosphorylation-dependent manner, potentially through the regulation of cytoskeletal dynamics.

### NME1 regulates the Hippo pathway through its histidine phosphorylation activity

**Figure 2.**
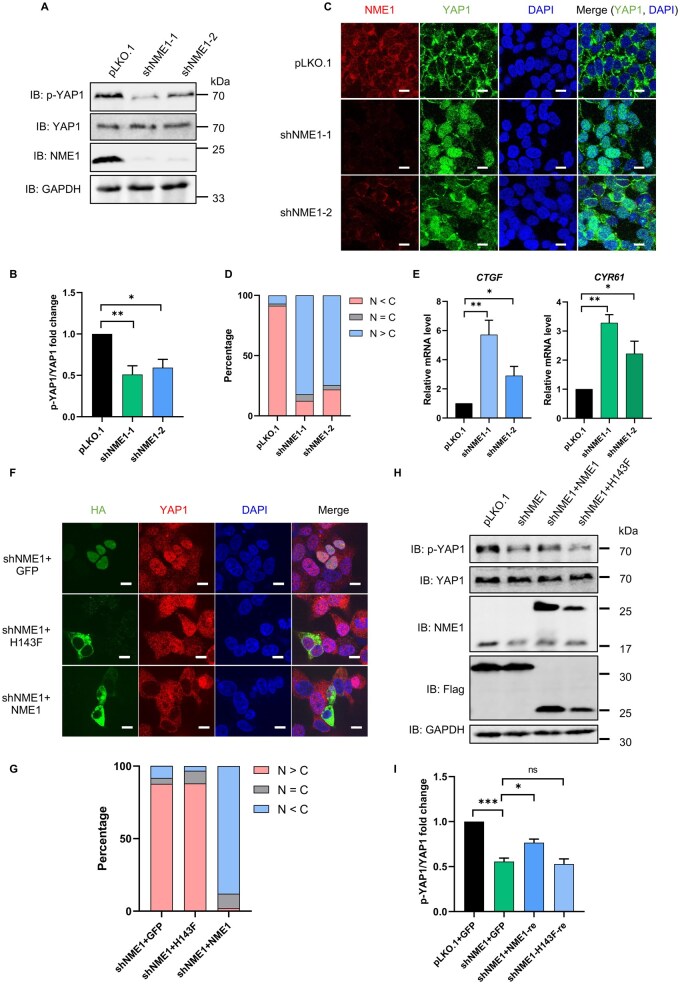
NME1 regulates Hippo pathway in a histidine phosphorylation-dependent manner. (A) Immunoblot analysis of pLKO.1, shNME1-1, and shNME1-2 cell lines in HEK293T. Proteins were separated by SDS-PAGE and probed with anti-p-YAP1(S127), anti-YAP1, anti-GAPDH (loading control), and anti-NME1 (knockdown efficiency). (B) Quantification of Fig. 2A grayscale values from three independent experiments (ImageJ). Data represent mean ± SEM; *P < 0.05, **P < 0.01 (Student’s *t*-test). (C) Immunofluorescence of YAP1 localization in pLKO.1 and shNME1 cells. NME1 (red), YAP1 (green), and DAPI (blue; nuclei). Merged images show YAP1/DAPI colocalization (scale bar: 10 μm). (D) Nuclear vs. cytoplasmic YAP1 distribution in pLKO.1 and shNME1 cells. N, nuclear YAP1; C, cytoplasmic YAP1. Fluorescence intensity was quantified in about 100 cells (ImageJ); bars show mean ± SEM (triplicates). (E) qPCR analysis of *CTGF* and *CYR61* mRNA in pLKO.1 vs. NME1 KD cells. Data represent mean ± SEM (triplicates); *P < 0.05, **P < 0.01 (Student’s *t*-test). (F) Immunofluorescence of YAP1 in NME1-knockdown (shNME1) cells overexpressing HA-GFP, HA-NME1, or HA-H143F. HA-tagged proteins are shown in green, YAP1 in red, and nuclei stained with DAPI in blue. Merged images show YAP1 nuclear localization relative to DAPI (scale bar: 10 μm). (G) Quantification of Fig. 2F: YAP1 N/C ratio in about 50 HA-positive cells (ImageJ). N, nuclear YAP1; C, cytoplasmic YAP1. (H) Immunoblot of pLKO.1 and NME1 KD cells overexpressing Flag-GFP, Flag-NME1, or Flag-H143F. Blots were probed with anti-p-YAP1(S127), anti-YAP1, anti-Flag (overexpression), anti-GAPDH (loading control), and anti-NME1 (to assess both knockdown efficiency and expression of exogenous NME1 or NME1-H143F). (I) Quantification of Fig. 2H grayscale values (three experiments; ImageJ). Data represent mean ± SEM; ns (not significant), *P < 0.05, ***P < 0.001 (Student’s *t*-test).

To investigate whether NME1 regulates the Hippo pathway, we generated NME1-knockdown HEK293T cell lines and assessed YAP1 phosphorylation status. Western blot analysis revealed that NME1 depletion significantly reduced YAP1 phosphorylation (p-YAP1 at Ser127), while total YAP levels remained unchanged (Fig. 2A and 2B). Immunofluorescence analysis further demonstrated that knockdown of NME1 led to increased nuclear localization of YAP1 (Fig. 2C and 2D). Consistent with YAP1 activation, qPCR analysis of YAP1 target genes (*CTGF*, *CYR61*) revealed a significant increase in their mRNA levels upon NME1 depletion, confirming that NME1 positively regulates the Hippo pathway (Fig. 2E). We found that NME1, but not NME2, regulates the Hippo signaling pathway (Fig. S1A and S1B).

To further validate the histidine phosphorylation-dependent function of NME1, we performed rescue experiments by expressing either wild-type NME1 or the kinase-deficient NME1-H143F mutant in NME1-knockdown cells. Immunofluorescence analysis showed that re-expression of wild-type NME1, but not NME1-H143F, restored YAP1 cytoplasmic localization (Fig. 2F and 2G). Correspondingly, Western blot analysis demonstrated that only wild-type NME1 re-expression rescued p-YAP1 levels in NME1-knockdown cells, whereas NME1-H143F failed to do so (Fig. 2H and 2I). These findings demonstrate that NME1 positively regulates the Hippo pathway in HEK293T cells and that this regulation depends on its histidine phosphorylation activity.

### ARHGAP17 functions downstream of NME1 in Hippo pathway

**Figure 3.**
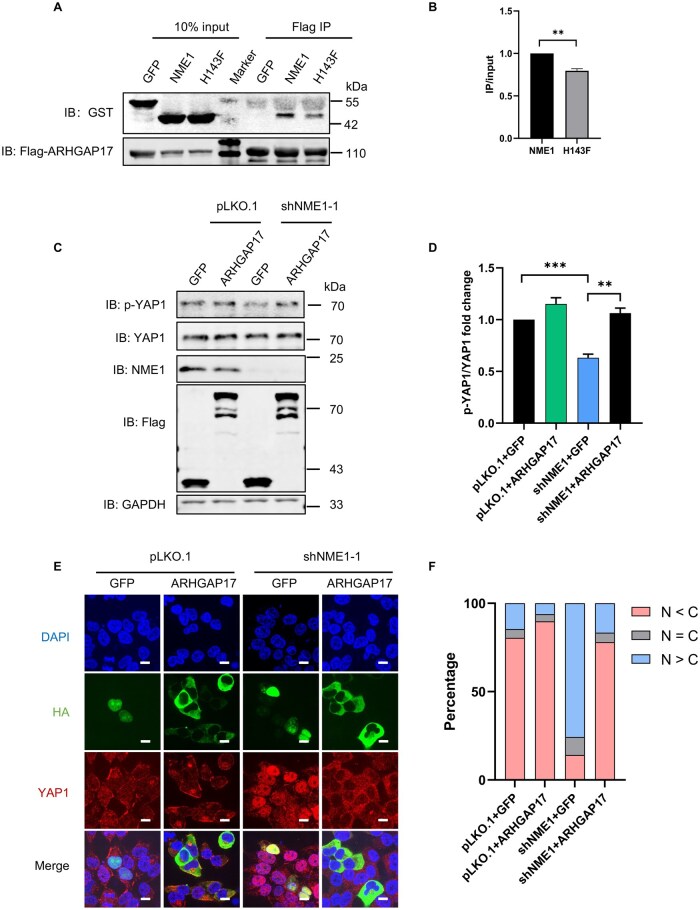
ARHGAP17 could act as a downstream effector of NME1 in the Hippo pathway. (A) Co-immunoprecipitation of NME1/NME1-H143F with ARHGAP17 in HEK293T cells. GST-tagged NME1/NME1-H143F and Flag-HA-tagged ARHGAP17 were co-expressed, with GST-GFP as negative control. (B) Quantification of Fig. 3A interaction strength from three independent experiments (ImageJ). Data represent mean ± SEM; **P < 0.01 (Student’s *t*-test). (C) Western blot analysis of Flag-ARHGAP17 overexpression in pLKO.1 and NME1 knockdown cells (Flag-GFP as control). Blots were probed with anti-p-YAP1(S127), anti-YAP1, anti-NME1, anti-Flag, and anti-GAPDH (loading control). (D) Quantification of Fig. 3C protein levels from three experiments (ImageJ). Data represent mean ± SEM; **P < 0.01, ***P < 0.001 (Student’s *t*-test). (E) Immunofluorescence of YAP1 localization in pLKO.1 and shNME1 cells overexpressing Flag-HA-GFP or ARHGAP17. YAP1 (red), HA-tagged protein (green), and DAPI (blue; nuclei). Merged images show all channels (scale bar: 10 μm). (F) Quantification of Fig. 3E: nuclear vs. cytoplasmic YAP1 distribution in about 50 HA-positive cells (ImageJ). N, nuclear YAP1; C, cytoplasmic YAP1.

PhastID analysis identified ARHGAP17, a CDC42-specific Rho GTPase-activating protein (RhoGAP), as a potential downstream effector of NME1 in Hippo pathway regulation. To investigate their potential interaction, we utilized AlphaFold2 to predict the structural interface between NME1 and ARHGAP17. The predicted model suggests a potential direct interaction between the two proteins. Notably, a hydrogen bond is predicted to form between H118 of NME1 (corresponding to H143 in the long transcript variant) and T879 of ARHGAP17 (Fig. S2A). To validate this interaction, we performed immunoprecipitation assays and confirmed that ARHGAP17 interacts with wild-type NME1, while the interaction was reduced with the kinase-deficient NME1-H143F mutant (Fig. 3A and 3B). These findings collectively suggest that histidine phosphorylation at H143 of NME1 is critical for stabilizing the NME1 and ARHGAP17 interaction. However, *in vitro* kinase assays showed that NME1 cannot phosphorylate ARHGAP17, indicating that ARHGAP17 is not a substrate of NME1 (Fig. S2B).

To investigate the functional role of ARHGAP17 in Hippo pathway, we overexpressed ARHGAP17 in both control and NME1-knockdown HEK293T cells. Western blot analysis showed that ARHGAP17 overexpression significantly increased YAP1 phosphorylation levels in NME1-knockdown cells, partially rescuing the reduced p-YAP1 levels observed upon NME1 depletion (Fig. 3C and 3D). Immunofluorescence analysis further showed that ARHGAP17 overexpression in NME1-knockdown cells promoted cytoplasmic retention of YAP1, similar to the subcellular localization pattern seen in control cells (Fig. 3E and 3F). These results indicate that ARHGAP17 mediates NME1-dependent activation of the Hippo pathway and suggest that ARHGAP17 functions downstream of NME1 to regulate YAP1 phosphorylation and subcellular localization in a histidine phosphorylation-dependent manner.

### NME1 modulates CDC42 activity in a histidine phosphorylation-dependent manner

**Figure 4.**
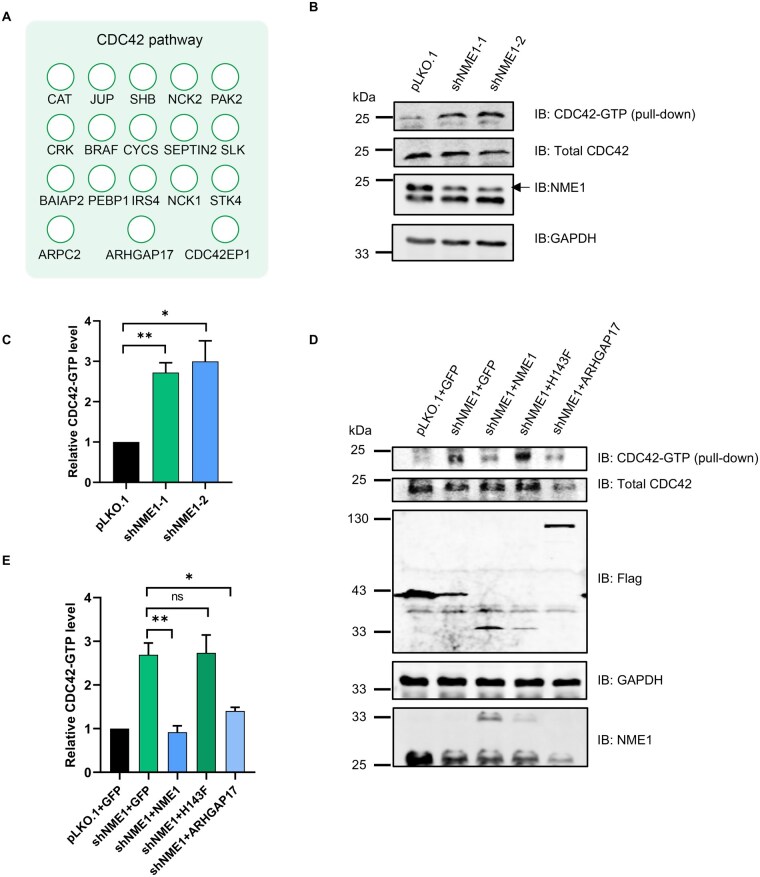
NME1 regulates CDC42 GTPase activity via ARHGAP17 in a histidine phosphorylation-dependent manner. (A) GO analysis of Ph-NME1 interactome identified significant enrichment for CDC42 pathway components. (B) Active CDC42 (CDC42-GTP) levels assessed by His-PBD pull-down in pLKO.1 vs. NME1 KD cells. Input lanes (5% total lysate) were probed with anti-CDC42 (total), anti-NME1 (knockdown efficiency), and anti-GAPDH (loading control). Pull-down fractions detected CDC42-GTP. (C) Quantification of Fig. 4B showing CDC42-GTP/total CDC42 ratio. Data represent mean ± SEM, *n* = 3; ImageJ; *P < 0.05, **P < 0.01 by Student’s *t*-test. (D) Rescue experiment: CDC42-GTP levels in NME1 KD cells expressing GFP (control), NME1, NME1-H143F, or ARHGAP17. Western blots were probed as in Figure B. (E) Quantification of Figure D demonstrating CDC42-GTP restoration. Data represent mean ± SEM, *n* = 3; ns (not significant), *P < 0.05, **P < 0.01 by Student’s *t*-test.

Proteomic analysis from the PhastID dataset revealed enrichment of the CDC42 signaling pathway in the NME1 interactome, suggesting a potential regulatory link between NME1 and CDC42 activity (Fig. 4A). Given that CDC42 is a well-known regulator of actin cytoskeleton dynamics and Hippo pathway activity, we investigated whether NME1 affects CDC42 activation. CDC42-GTP levels were assessed using a pull-down assay with His-PBD beads, which specifically capture active GTP-bound CDC42 [18]. We validated that His-PBD specifically pulled down constitutively active CDC42-G12V, but not inactive CDC42-T17N (Fig. S3). We observed that NME1 depletion led to a significant increase in CDC42-GTP levels, suggesting that NME1 negatively regulates CDC42 activity (Fig. 4B and 4C).

To determine whether this regulation depends on NME1’s histidine phosphorylation activity, we performed rescue experiments by re-expressing either wild-type NME1 or the kinase-deficient NME1-H143F mutant in NME1-knockdown cells. Re-expression of wild-type NME1, but not NME1-H143F, restored CDC42-GTP levels to baseline, reinforcing the role of histidine kinase activity in this regulation (Fig. 4D and 4E). Notably, overexpression of ARHGAP17 also rescued CDC42-GTP levels in NME1-knockdown cells, supporting ARHGAP17’s role as a mediator of NME1-dependent CDC42 inactivation. Collectively, these findings demonstrate that NME1 modulates CDC42 activity in a histidine phosphorylation-dependent manner, potentially linking NME1 to the regulation of cytoskeletal organization and Hippo pathway activity through CDC42 signaling.

### NME1 modulates the Hippo pathway through the CDC42–PAK–cytoskeleton axis

**Figure 5.**
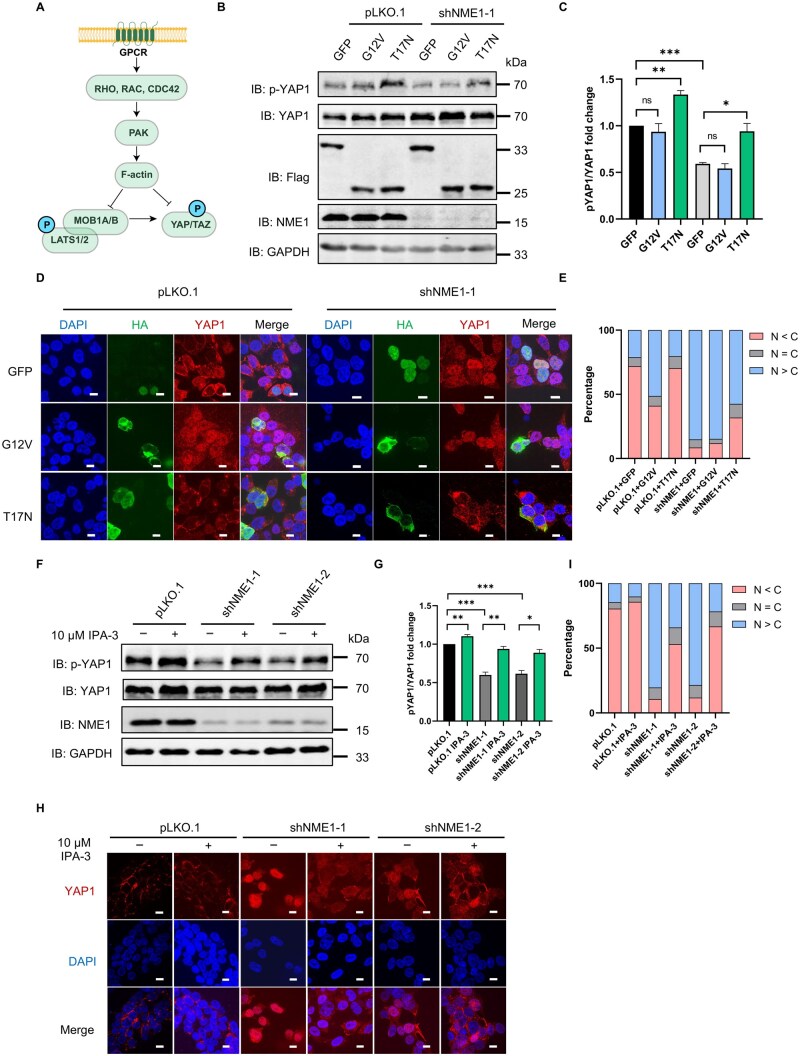
NME1 regulates Hippo signaling pathway through CDC42–PAK–F-actin axis. (A) Schematic of Rho family members transducing GPCR signals to regulate F-actin via PAK, ultimately influencing YAP phosphorylation. (B) Western blot analysis of p-YAP1 levels in total cell lysates from stable HEK293T cell lines (pLKO.1 control or NME1 knockdown) following transient transfection with plasmids expressing GFP, CDC42-G12V (constitutively active), or CDC42-T17N (dominant-negative). Blots were probed with anti-p-YAP1, anti-YAP (total), anti-Flag (overexpression), anti-NME1, and anti-GAPDH (loading control). (C) Quantification of Fig. 5B from three independent experiments (ImageJ). Data represent mean ± SEM; *P < 0.05, **P < 0.01, ***P < 0.001 (Student’s *t*-test). (D) Immunofluorescence of YAP1 localization in cells from HEK293T pLKO.1 and NME1 knockdown lines overexpressing GFP, CDC42-G12V (constitutively active), or CDC42-T17N (dominant-negative). YAP1 (red), HA-tagged proteins (green), and DAPI (blue; nuclei). Merged images show all channels (scale bar: 10 μm). (E) Quantification of Fig. 5D: nuclear to cytoplasmic YAP1 ratio was calculated from about 50 individually analyzed HA-positive cells (ImageJ). N, nuclear YAP1; C, cytoplasmic YAP1. (F) Western blot analysis of p-YAP1 levels in HEK293T pLKO.1, shNME1-1, and shNME1-2 cells treated with DMSO or 10 μmol/L IPA-3 for 24 h. Blots were probed with anti-p-YAP1, anti-YAP, anti-NME1, and anti-GAPDH (loading control). (G) Quantification of Fig. 5F from three independent experiments (ImageJ). Data represent mean ± SEM; *P < 0.05, **P < 0.01, ***P < 0.001 (Student’s *t*-test). (H) Immunofluorescence of YAP1 localization in cells from Fig. 5F. YAP1 (red) and DAPI (blue; nuclei). Merged images show both channels (scale bar: 10 μm). (I) Quantification of Figure H: nuclear vs. cytoplasic YAP1 distribution in about 100 cells (ImageJ). N, nuclear YAP1; C, cytoplasmic YAP1.

When receiving signals from GPCRs (G protein-coupled receptors), Rho family members (Rho, Rac, CDC42) regulate F-actin by modulating PAK, and then inhibit YAP1 phosphorylation directly or indirectly (Fig. 5A). To examine the role of CDC42 activity in Hippo pathway regulation under NME1 knockdown, we overexpressed the constitutively active CDC42-G12V mutant or the dominant-negative CDC42-T17N mutant in control and NME1-knockdown HEK293T cells. Western blot analysis revealed that overexpression of CDC42-G12V resulted in a modest reduction in p-YAP1 (Ser127) levels, although the change did not reach statistical significance—likely due to variability in transfection efficiency (Fig. 5B and 5C). In contrast, CDC42-T17N expression significantly restored p-YAP1 levels in both control and NME1-knockdown cells. Immunofluorescence with higher sensitivity and resolution further showed that overexpression of CDC42-G12V promoted nuclear translocation of YAP1, while CDC42-T17N caused cytoplasmic retention of YAP1 in NME1-knockdown cells (Fig. 5D and 5E). These results support the role of active CDC42 in inhibiting the Hippo pathway by reducing YAP1 phosphorylation and facilitating its nuclear translocation, while inactive CDC42 has the opposite effect.

To further investigate the downstream effector pathway, we treated cells with IPA-3, a selective, ATP non-competitive PAK1 inhibitor [19]. IPA-3 treatment significantly increased p-YAP1 levels in both control and NME1-knockdown cells (Fig. 5F and 5G), and promoted YAP1 cytoplasmic retention as shown by immunofluorescence (Fig. 5H and 5I). Consistently, we found that NME1-knockdown cells exhibited significant cytoskeletal remodeling with increased pseudopodia formation (Fig. S4A and S4B). These findings indicate that PAK acts downstream of CDC42 to mediate Hippo pathway suppression, with its inhibition restoring YAP1 phosphorylation even in NME1-depleted conditions. To test whether NME1 functions upstream of CDC42, we examined p-YAP1 levels in CDC42-knockdown cells overexpressing either wild-type NME1 or its kinase-deficient mutant NME1-H143F. Notably, NME1-H143F failed to reverse the elevated p-YAP1 levels in CDC42-knockdown cells (Fig. S4C), indicating that the regulatory effect of NME1 on the Hippo pathway is CDC42-dependent.

**Figure 6.**
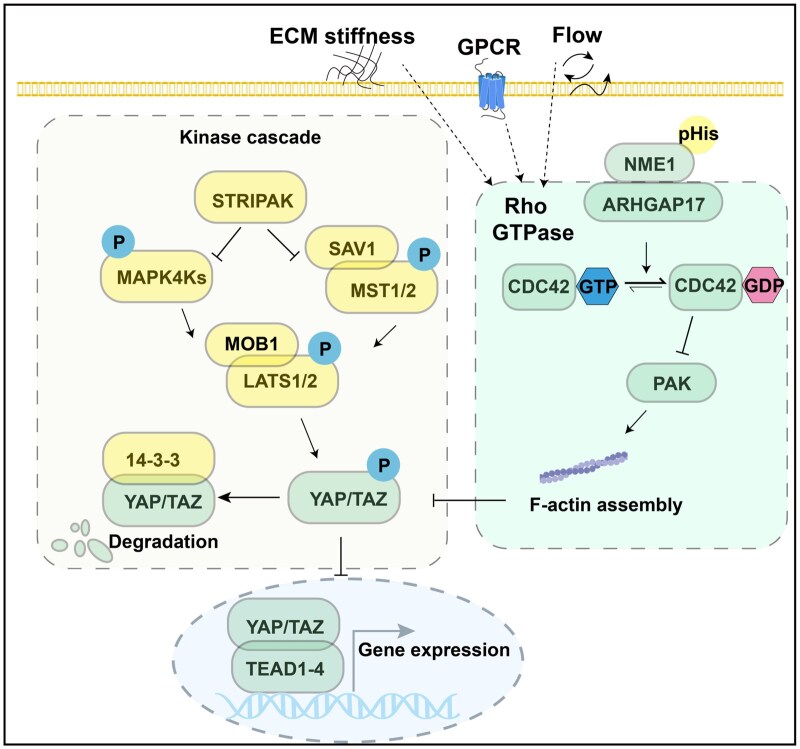
NME1 regulates Hippo pathway activity via ARHGAP17–CDC42–PAK–cytoskeleton axis. Mechanical cues such as extracellular matrix (ECM) stiffness, GPCR signaling, and flow regulate the Hippo pathway through both canonical kinase cascades (left) and Rho GTPase-mediated cytoskeletal remodeling (right). NME1 promotes histidine phosphorylation-dependent interaction with ARHGAP17, a CDC42-specific GTPase-activating protein (GAP), leading to increased CDC42-GTP levels. Activated CDC42 engages PAK to regulate F-actin dynamics, which modulate YAP/TAZ phosphorylation and localization. Canonical Hippo kinases (MST1/2, LATS1/2) phosphorylate and inhibit YAP/TAZ, preventing their nuclear accumulation and TEAD-mediated gene transcription. This model highlights the role of NME1 as an upstream regulator linking cytoskeletal remodeling to Hippo signaling.

Collectively, these results demonstrate that NME1 regulates the Hippo pathway by modulating the ARHGAP17–CDC42–PAK–cytoskeleton axis in a histidine phosphorylation activity-dependent manner, linking cytoskeletal organization to Hippo pathway activation and providing mechanistic insight into how NME1 functions as a metastasis suppressor (Fig. 6).

## Discussion

Histidine phosphorylation remains a largely underexplored post-translational modification in mammalian cells due to its chemical instability and lack of detection tools [1]. In this study, using PhastID-based proximity labeling, we uncovered a previously unrecognized role of NME1 in regulating the Hippo pathway through its histidine phosphorylation activity. We demonstrate that NME1 modulates CDC42 activity via ARHGAP17, a CDC42-specific RhoGAP, thereby influencing cytoskeletal dynamics and Hippo pathway activation. These findings establish the NME1–ARHGAP17–CDC42–cytoskeleton axis as a critical regulatory mechanism of Hippo signaling and provide new insights into the functional repertoire of NME1.

The Hippo pathway is crucial for regulating organ size, cell proliferation, and apoptosis, with dysregulation contributing to cancer progression. While previous studies have highlighted the involvement of Rho family GTPases in Hippo regulation, the contribution of histidine phosphorylation to this regulation has remained unclear. Our findings suggest that NME1, through its histidine kinase activity, modulates Hippo pathway activity by influencing cytoskeletal dynamics, consistent with the established role of mechanical cues in Hippo pathway regulation. Given that NME1 can function both as a NDPK and a histidine kinase, it may influence local GTP levels near key signaling molecules, affecting their activity. This dual function suggests a possible role for NME1 in coordinating metabolic status and cytoskeletal signaling, further reinforcing its importance in cellular homeostasis.

Our study identifies ARHGAP17 as a downstream effector of NME1, linking histidine phosphorylation to CDC42 inactivation. ARHGAP17, known for its role in cytoskeletal organization and junctional integrity, exhibited reduced interaction with the kinase-deficient NME1-H143F mutant, highlighting the functional relevance of NME1’s histidine phosphorylation in this regulatory axis. Given ARHGAP17’s role in Hippo pathway regulation, it likely serves as a key mediator linking NME1’s histidine phosphorylation activity to YAP/TAZ regulation. The interplay between GTPase signaling and Hippo pathway activity has been well-documented, with CDC42 and RhoA acting as upstream modulators [20, 21]. The precise mechanism by which NME1 regulates ARHGAP17 remains to be fully elucidated. Current findings indicate that ARHGAP17 is not a direct phosphorylation substrate of NME1, suggesting alternative regulatory mechanisms may be involved. One possibility is that NME1 physically interacts with ARHGAP17 to enhance its GTP hydrolysis activity toward CDC42-GTP. Alternatively, NME1 might modulate ARHGAP17–CDC42 activity through intermediary proteins. Another potential mechanism involves NME1 altering local GTP availability via its NDPK activity, thereby influencing the ARHGAP17–CDC42 axis. These hypotheses require further experimental validation. Overall, our findings suggest that histidine phosphorylation may regulate this axis, adding a new layer of complexity to the existing model of Hippo pathway control.

Our data further suggest that NME1 indirectly modulates this process through the CDC42–PAK axis. CDC42 regulates actin polymerization through PAK, which affects cellular tension and Hippo pathway activation [22–24]. We show that NME1 depletion increases CDC42-GTP levels, leading to reduced YAP phosphorylation and enhanced nuclear localization, while restoration of NME1 or ARHGAP17 rescues this phenotype. Inhibition of PAK with IPA-3 restored YAP phosphorylation and cytoplasmic localization in NME1-knockdown cells, confirming the downstream role of the CDC42–PAK pathway in mediating Hippo pathway suppression. These findings suggest that histidine phosphorylation by NME1 fine-tunes cytoskeletal dynamics to control Hippo pathway activity.

NME1 has long been recognized as a tumor metastasis suppressor, but its precise molecular function has remained elusive. Our study connects NME1’s histidine kinase activity with Hippo pathway regulation, suggesting that NME1 loss may contribute to tumor progression through dysregulated mechanotransduction and promoting YAP activation. The Hippo pathway is frequently disrupted in cancers such as lung, breast, and colorectal cancer, where YAP hyperactivation drives uncontrolled proliferation and metastasis. Given that CDC42 is often hyperactivated in invasive tumors, targeting the NME1–ARHGAP17–CDC42 axis could provide a novel therapeutic strategy. Furthermore, histidine phosphorylation has been implicated in metabolic control, raising the possibility that NME1-mediated Hippo regulation may be linked to metabolic reprogramming in cancer cells. Exploring this connection may reveal new therapeutic targets for treating tumors with Hippo pathway dysregulation.

In summary, our study identifies NME1 as a novel upstream regulator of the Hippo pathway, acting through the histidine phosphorylation-dependent NME1–ARHGAP17–CDC42–cytoskeleton axis. Our findings suggest that histidine phosphorylation plays a previously unrecognized role in cytoskeletal signaling and Hippo pathway control. Given the involvement of Hippo signaling in cancer and mechanotransduction, our results highlight a potential therapeutic target for tumors with Hippo pathway dysregulation. Further research into histidine phosphorylation-dependent signaling may reveal additional regulatory mechanisms with broad implications for cellular homeostasis and disease progression.

## Research limitations

This study has several limitations. First, while we employed proximity labeling technology to investigate the proximal protein networks of NME1 and NME1-H143F, the extended biotinylation duration may have increased background signals. Future work optimizing labeling duration could improve data specificity and quality. Second, we have not fully elucidated the mechanistic details of how NME1 regulates ARHGAP17 to modulate CDC42 activity. Structural studies to identify specific interaction domains between NME1 and ARHGAP17 will be important to elucidating their precise regulatory mechanisms. Third, we did not comprehensively explore the downstream phenotypic consequences of NME1-mediated YAP1 regulation. Whether and how this regulatory axis affects tumor cell migration, invasion, and tumorigenesis remain to be determined in future studies.

## Materials and methods

### Research ethics

No human or animal subjects were involved in this research. The cell lines were obtained from American Type Culture Collection (ATCC)/other repository and were maintained according to the supplier’s recommendations. Because no original human or animal tissue was procured and no live vertebrates were used, ethical approval was not required for this work.

### Cell culture and transfection

HEK293T cells were obtained from the ATCC and maintained in DMEM supplemented with 10% fetal bovine serum at 37 °C in a humidified 5% CO_2_ atmosphere. For transient transfection, cells were transfected with polyethylenimine (PEI) at a PEI:plasmid ratio of 3.3:1 and harvested 48 h post-transfection. For stable transfection, cells were infected with lentivirus and selected with appropriate antibiotics.

### Proximity labeling and LC–MS/MS analysis

For proximity labeling, cells were incubated with 50 µmol/L biotin for 16 h before harvesting. Cells were lysed in buffer containing 10 mmol/L HEPES (pH 7.9), 1.5 mmol/L MgCl_2_, 10 mmol/L KCl, 0.5% NP-40, and protease inhibitor cocktail. Streptavidin magnetic beads were used to capture biotinylated proteins at 4°C for 2 h. Beads were washed sequentially with RIPA buffer, 2 mol/L urea in 10 mmol/L Tris–HCl (pH 8.0), 2% Sodium Dodecyl Sulfate–Polyacrylamide Gel Electrophoresis (SDS-PAGE), Tris-Buffered Saline with Tween 20 (TBST), and Tris-Buffered Saline (TBS). Proteins were reduced with 4 mmol/L Dithiothreitol (DTT), alkylated with 10 mmol/L Iodoacetamide (IAA), and digested with trypsin overnight at 37°C. Peptides were desalted using C18 tips and analyzed by Liquid Chromatography-Tandem Mass Spectrometry (LC–MS/MS).

### Western blotting

Protein concentrations were measured using a Bicinchoninic Acid Assay (BCA) assay (Thermo Fisher). Samples were mixed with SDS loading buffer and boiled for 10 min. An unboiled control group was also included for comparison. Samples were separated by SDS-PAGE, then transferred to nitrocellulose membranes and blocked with 5% Bovine Serum Albumin (BSA) for 1 h at room temperature. Membranes were incubated overnight at 4°C with primary antibodies, followed by fluorescent secondary antibodies (LI-COR) for 1 h at room temperature. Blots were visualized using the Odyssey Imaging System. (The antibody list is provided in Table S1.)

### Immunofluorescence

Cells grown on coverslips were fixed with 4% paraformaldehyde for 20 min on ice and permeabilized with 0.1% Triton X-100 for 15 min. After blocking with 5% goat serum for 1 h, cells were incubated with primary antibodies (HA, YAP1, Streptavidin-AF488, Phalloidin-488) for 1 h, followed by secondary antibodies for 1 h in the dark. Nuclei were stained with DAPI. Fluorescence images were acquired using a Leica TCS SP8 or Nikon AX confocal microscope, and YAP subcellular localization was analyzed with ImageJ.

### Real-time PCR

Total RNA was extracted using TRIzol reagent, and cDNA was synthesized using PrimeScript™ RT reagent Kit (Takara). Quantitative PCR was performed using SYBR Green Master Mix with GAPDH as an internal control. Primer sequences:

*GAPDH*,

5′-GATTCCACCCATGGCAAATTC-3′ (forward),

5′-CTGGAAGATGGTGATGGGATT-3′ (reverse).

*CTGF*,

5′-CCAATGACAACGCCTCCTG-3′ (forward),

5′-TGGTGCAGCCAGAAAGCTC-3′ (reverse).

*CYR61*,

5′-AGCCTCGCATCCTATACAACC-3′ (forward),

5′-TTCTTTCACAAGGCGGCACTC-3′ (reverse).

### CDC42-GTP pulldown assay

Cells were lysed in NETN buffer (50 mmol/L Tris-HCl, pH 7.5, 20 mmol/L NaCl, 0.5% NP-40, 10% glycerol, 0.1 mmol/L DTT, 2 mmol/L MgCl_2_). Equal protein amounts were incubated with bacterially expressed His-PBD beads at 4°C for 2 h to capture active CDC42-GTP. Beads were washed, and bound proteins were analyzed by western blotting.

### Plasmids and mutagenesis

sh*NME1*-1: 5′-CCGCCTTGTTGGTCTGAAATT-3′.

sh*NME1*-2: 5′-TCCGCCTTGTTGGTCTGAAAT-3′.

*NME1*-H143F: 5′- AGGAACATTATATTTGGC-3′ (forward) and

5′- AGAATCACTGCCAAATAT-3′ (reverse).

*NME1*-rescue: 5′-TTAGTTGGACTGAAATTCATGCAAG-3′ (forward) and

5′-TCCAACTAAAGAGAATCCTTTCTG-3′ (reverse).

*CDC42*-G12V: 5′-TGTTGTGGGCGATGTTGCT-3′ (forward) and

5′-AGACATGTTTTACCAACAGCAACATC-3′ (reverse).

*CDC42*-T17N: 5′-CGATGGTGCTGTTGGTAAAAACTGT-3′ (forward) and

5′-TGTTGTGTAGGATATCAGGAGACAGTTTTT-3′ (reverse).

### Statistical analysis

All quantitative experiments were performed with three independent biological repeats. The results were analyzed and plotted using GraphPad Prism 9 software. All data are presented as mean ± standard error of the mean (SEM). Statistical analysis was performed using unpaired two-tailed Student’s *t*-test (*P < 0.05; **P < 0.01, and ***P < 0.001). A *P*-value < 0.05 was considered statistically significant.

## Supplementary Material

lnag002_Supplementary_Data

## Data Availability

All data needed to evaluate the conclusion in this paper are present in the paper and/or in the Supplementary Material.
