# Supplementary material for: Histidine phosphorylation of NME1 regulates the Hippo pathway via the ARHGAP17–CDC42–cytoskeleton axis
Source: Life Med. 2026 Mar 12;5(1):lnag002. doi: 10.1093/lifemedi/lnag002 (PMC13070683; doi:10.1093/lifemedi/lnag002)
Supplement: lnag002_Supplementary_Data [file lnag002_supplementary_data.zip › Histidine phosphorylation of NME1_SI.docx]

**Histidine phosphorylation of NME1 regulates the Hippo pathway via the ARHGAP17–CDC42–cytoskeleton axis**

Xian Liu^1,#^ , Zhongnan Chen^1,#^, Jianxi Zhu^1^, Shengcheng Deng^1^, Zhifen Zhou^2,3,^*, Wenbin Ma^1,^*, Zhou Songyang^1,2,3,^*

^1^MOE Key Laboratory of Gene Function and Regulation, State Key Laboratory of Biocontrol, Guangzhou Key Laboratory of Healthy Aging Research, School of Life Sciences, Institute of Healthy Aging Research, Sun Yat-sen University, Guangzhou 510275, China

^2^Innovative Center of Health, Longevity and Synthetic Biology, Hainan Academy of Medical Sciences, Hainan Medical University, Haikou 571199, China

^3^Sun Yat-sen Memorial Hospital, Sun Yat-sen University, Guangzhou 510120, China

^#^These authors contributed equally to this work.

*Correspondence: [songyanz@mail.sysu.edu.cn](mailto:songyanz@mail.sysu.edu.cn) (Z.S.), [mawenbin@mail.sysu.edu.cn](mailto:mawenbin@mail.sysu.edu.cn) (W.M.), [zhzhfen@alumni.sysu.edu.cn](mailto:zhzhfen@alumni.sysu.edu.cn) (Z.Z.)

**Figure S1. NME1, but not NME2, regulates the Hippo pathway.**

(A) Immunofluorescence images showing YAP1 localization. Green channel: YAP1 signal; blue channel, DAPI (nuclear) signal; Merge, overlay of both channels. Scale bar: 10 μm.

(B) Quantitative analysis of images from Fig. S1A. Approximately 100 cells per group were analyzed using ImageJ software to measure YAP1 fluorescence intensity in nuclear and cytoplasmic regions.

**Figure S2. Structural prediction of the NME1–ARHGAP17 interaction and assessment of ARHGAP17 phosphorylation by NME1.**

(A) Predicted complex structure of NME1 (yellow) and ARHGAP17 (blue), generated using AlphaFold2 and visualized with ChimeraX software. The left panel shows the overall structure of the complex. The right panel provides a magnified view of the interaction interface, highlighting a hydrogen bond (pink dashed line) formed between the His118 residue of NME1 and the Thr879 residue of ARHGAP17.

(B) *In vitro* kinase assay using [γ-^32^P] ATP labeling was performed to detect NME1-mediated phosphorylation of ARHGAP17. Flag-GFP and Flag-NME1-H143F purified from HEK293T cells served as negative controls. Autoradiography indicated [γ-^32^P] ATP signals, while standard Western blot with anti-Flag antibody confirmed protein loading.

**Figure S3. Test of His-PBD pull-down efficiency.**

Western blot analysis of His-PBD pull-down efficiency. GFP served as the control, while T17N (inactive CDC42) and G14V (active CDC42) were used as experimental groups. Proteins were detected using anti-CDC42, anti-Flag, and anti-His antibodies.

**Figure S4. NME1 affects F-actin architecture.**

(A) Immunofluorescence staining of F-actin in HEK293T cell lines (pLKO.1 control, sh*NME1*-1, and sh*NME1*-2 knockdown groups). F-actin was labeled with phalloidin (green), and nuclei were stained with DAPI (blue). White arrows indicate representative filopodia structures. Scale bar: 10 μm. (B) Quantification of filopodia formation rate in pLKO.1, shNME1-1, and shNME1-2 groups (*n* ≈ 100 cells per group). (C) Immunoblot analysis was performed on pLKO.1 and shCDC42 cell lines in HEK293T cells. Both cell lines were transfected to overexpress their respective Flag-HA-tagged proteins. Proteins were separated by SDS-PAGE and subsequently probed with antibodies against p-YAP1 (S127), YAP1, GAPDH (loading control), Flag (to detect overexpressed proteins), and CDC42 (to assess knockdown efficiency).
