# Supplementary figures and images for: Histidine phosphorylation of NME1 regulates the Hippo pathway via the ARHGAP17–CDC42–cytoskeleton axis
Source: Life Med. 2026 Mar 12;5(1):lnag002. doi: 10.1093/lifemedi/lnag002 (PMC13070683; doi:10.1093/lifemedi/lnag002)

Figure S1

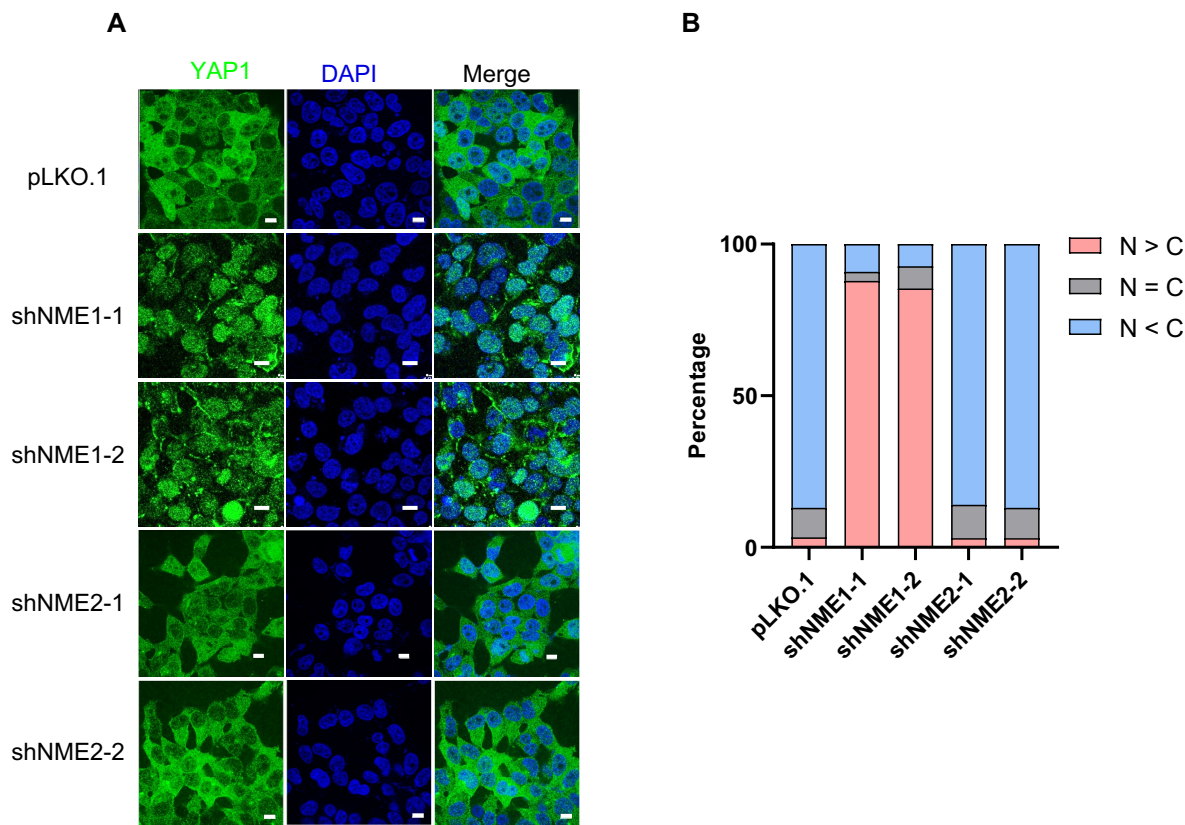

Figure S2

A

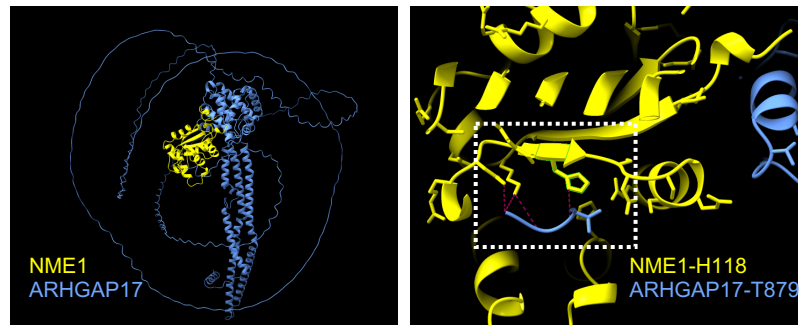

B

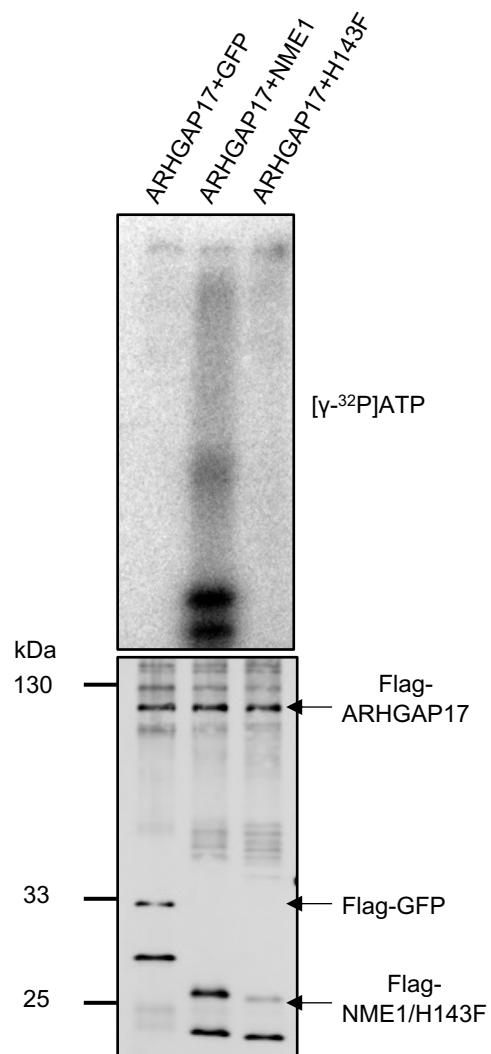

**Figure S3**

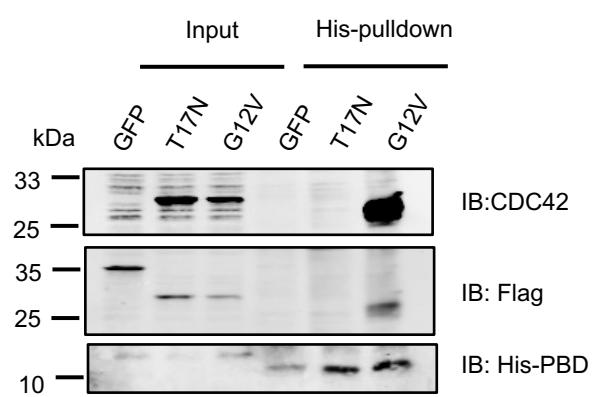

**Figure S4**

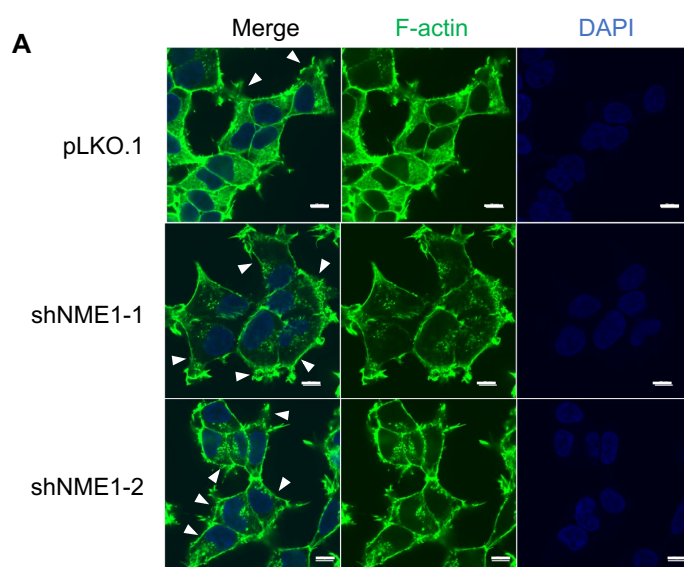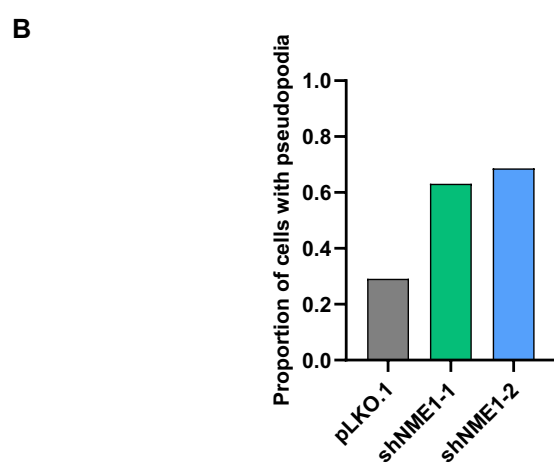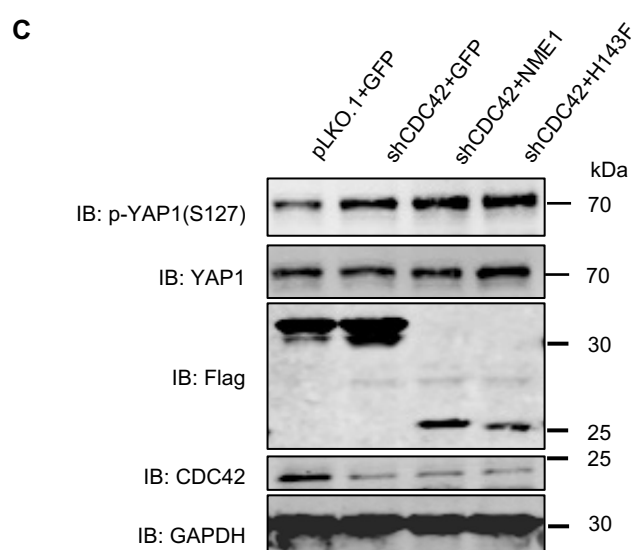

Supplement: lnag002_Supplementary_Data [file lnag002_supplementary_data.zip › Figures of NME1_PE_SI.pdf]
